# Epidemiology and clinical features of viral anterior uveitis in southern Taiwan—diagnosis with polymerase chain reaction

**DOI:** 10.1186/s12886-019-1093-2

**Published:** 2019-04-03

**Authors:** Yu-Ting Hsiao, Ming-Tse Kuo, Wei-Yu Chiang, Tsai-Ling Chao, Hsi-Kung Kuo

**Affiliations:** 1 Department of Ophthalmology, Kaohsiung Chang Gung Memorial Hospital, Kaohsiung, Taiwan; 2 Department of Ophthalmology, Kaohsiung Chang Gung Memorial Hospital and Chang Gung University College of Medicine, No.123, Dapi Rd., Niaosong Dist, Kaohsiung, 833 Taiwan; 3 Department of Laboratory Medicine, Kaohsiung Chang Gung Memorial Hospital and Chang Gung University College of Medicine, Kaohsiung, Taiwan

**Keywords:** Polymerase chain reaction, Anterior uveitis, Virus, Cytomegalovirus, Hypertensive uveitis

## Abstract

**Background:**

To report the epidemiology and clinical features of viral anterior uveitis in patients in southern Taiwan.

**Methods:**

A retrospective, case series study. HLA-B27 negative anterior uveitis patients with increased intraocular pressure or corneal edema seen at Kaohsiung Chang Gung Memorial Hospital from January 1, 2007 to January 31, 2018 had their aqueous sent for polymerase chain reaction analysis. Their records were reviewed for demographic data, ocular findings, and laboratory results.

**Results:**

In the aqueous samples obtained from 102 eligible eyes, 42 eyes were herpesviridae-positive, which included 9 with herpes simplex virus (8.8%), 5 with varicella-zoster virus (4.9%), 27 with cytomegalovirus (26.5%), and 1 with Epstein-Barr virus (1%). Herpesviridae-positive patients were more likely to be male, and have glaucoma. Glaucoma and pseudophakic eyes were significantly associated with CMV-positive eyes.

**Conclusion:**

PCR analysis of the anterior chamber fluid is important for the confirmation of the diagnosis of viral anterior uveitis. Cytomegalovirus anterior uveitis is not uncommon in patients in southern Taiwan, and it may follow an uneventful cataract extraction in immunocompetent patients.

## Background

Uveitis is a sight-threatening inflammatory ocular disease, and leads to 5–10% of visual impairment worldwide [1, 2]. Globally, uveitis and its associated complications account for up to 25% of blindness in developing countries, and varies from 3 to 10% in developed countries in both Europe and the United States [3–5]. Anterior uveitis (AU) is the most common form of uveitis in most regions of the world, representing between 28 and 50% of all uveitis cases in Asian countries such as Japan, Korea, and India [6].

HLA-B27 acute anterior uveitis (AAU) accounts for between 6 and 13% of all anterior uveitis in Asia [7]. HLA-B27 AAU is characterized by association with systemic diseases, particularly ankylosing spondylitis, male prevalence, hypopyon, unilateral or alternating bilateral involvement, fibrinous reaction, and papillitis [8]. In patients with HLA-B27 negative anterior uveitis, cases with hypertensive anterior uveitis are commonly associated with Posner-Schlossman syndrome (PSS), or a viral infection due to herpes simplex virus (HSV), varicella-zoster virus (VZV), or cytomegalovirus (CMV) [7, 9–11]. Non-HLA associated AU patients with relatively normal intraocular pressure (IOP) are classified as Fuchs’ heterochromic iridocyclitis (FHI), rubella virus infection, or idiopathic [10, 12].

Herpetic AU due to either HSV or VZV infection is a relatively common condition and currently accounts for 5 to 10% of all uveitis cases seen at tertiary referral centers [13]. In the last decade, due to the polymerase chain reaction (PCR) being used in viral detection of many ocular inflammations, CMV has also been regarded as a common cause of AU [13–15]. However, there is a wide range of clinical presentations of CMV AU [16]. It may present as a chronic anterior uveitis or as a recurrent episodic iritis with raised IOP resembling PSS [17]. HSV and VZV are considered the most common causes of infectious AU in the Western countries [18, 19], but CMV has been reported to be more predominant among the Chinese and Japanese [17, 20]. Some studies have presented some characteristic clinical findings that may enable the physician to make a clinical diagnosis and differentiation between various viral-induced infections [19, 20]. Of patients with AU caused by HSV, VZV or CMV, VZV AU patients had severe aqueous flare and the highest viral load in the aqueous humor, while CMV AU patients had the mildest intraocular inflammation, lowest corneal endothelial cell density, and highest IOP [20]. Although the AU caused by the three herpetic viruses may have some distinct features, a definitive diagnosis can only be made by anterior chamber fluid analysis at present [21].

There are relatively few studies on the epidemiology and clinical characteristics of viral anterior uveitis in Taiwan although this disease could cause a devastating loss of quality of life and productivity to those affected [22, 23]. The purpose of this study is to evaluate the percentage of virus-associated AU in non-HLA associated AU in southern Taiwan. In this article, we also compared the characteristics of CMV-positive eyes and herpesviridae-negative anterior uveitis eyes to determine if there were any signs or factors that could allow the physician to differentiate between them without having to resort to aqueous tapping.

## Methods

### Patients

This was a retrospective review of patients with anterior uveitis who were investigated and managed according to a standard protocol. The study adhered to the declaration of Helsinki and was approved by the Institutional Review Board of Chang Gung Memorial Hospital (study reference number: 201701609B0). All patients seen at Kaohsiung Chang Gung Memorial Hospital from January 1, 2007 to January 31, 2018 with uveitis had their aqueous humor samples sent for PCR analysis following informed consent. HLA-B27 negative anterior uveitis patients with increased IOP or corneal edema at diagnosis were included. Patients were excluded if they had intermediate, posterior or pan-uveitis, or had herpetic corneal epitheliopathy. In total, 102 eyes of 102 patients were recorded.

Patients’ records were reviewed for demographic data and clinical features. Main outcome measures were age, gender, laterality, visual acuity, intraocular pressure at diagnosis, presence of cornea edema, keratic precipitates, iris atrophy, lens status, glaucoma, corneal endothelial cell count and changes, and PCR results. Of patients with bilateral eye lesions, the eye with the worse status was chosen.

The diagnosis of glaucoma was made based on a combination of ocular hypertension and optic disc changes [24]. The endothelial cell count was measured by using a Topcon SP-3000P non-contact specular microscope (Topcon America Corporation, Paramus, NJ, USA) at the time of diagnosis. The relative endothelial cell loss was defined as the difference in endothelial cell count between the non-attack eye and the attack eye.

### Aqueous humor analysis, DNA extraction and amplification by PCR

An anterior chamber paracentesis was performed for all patients during an active episode under aseptic technique with the aid of a microscope after obtaining informed consent. At least 100 μL aqueous humor was aspirated and sent for PCR analysis. The collected samples were first frozen at − 20 °C and delivered to the laboratory in cold within 3 h. After mixing an aliquot of 100 μL aqueous humor with 1 mL normal saline, the sample was centrifuged at 13,200×g for 10 min in a microcentrifuge tube. The DNA in the precipitate was extracted using a commercial kit (QIAamp Viral RNA Mini Kit, Qiagen, Germany), and then stored at − 80 °C.

The extracted DNA was amplified by using 4 pairs of primers: one was used to amplify the long unique region 30 *(UL30)* gene of HSV-1 and HSV-2 (forward, 5′-CTGTTCTTCGTCAAGGCTCAC-3′; reverse, 5′- ATCGTCGTAAAACAGCAGGTC-3′) [25], the second was used to amplify the open reading frame 28 *(ORF28)* gene of VZV (forward, 5′- CGGCTCTGTTTTGTCCTC-3′; reverse, 5′-CTTCCCCACACCGTTTAC-3′) [25], the third was used to amplify the late antigen *gp64* gene of CMV (forward, 5′-CCGCAACCTGGTGCCCATGG-3′; 5′-CGTTTGGGTTGCGCAGCGGG-3′) [26], and the fourth was used to amplify the Epstein-Barr virus (EBV) nuclear antigen 2 *(EBNA2)* gene of Epstein-Barr virus (EBV; forward, 5′- TGGAAACCCGTCACTCTC -3′; 5′- TAATGGCATAGGTGGAATG -3′) [27]. The PCR mixture (25 μL) consisted of 2.5 μL of template DNA, 0.4 μM of each primer, and other necessary reagents from a PCR kit (JMR-THS5; JMR Holdings, Inc., St. Augustine, FL, USA). PCR amplification was performed in an automated thermocycler (GeneAmp PCR system 2700, Applied Biosystems, Foster City, CA) under the following cycling conditions: initial denaturation (95 °C for 3 min), 35 cycles of denaturation (95 °C for 30 s), annealing (55 °C for 45 s), extension (72 °C for 45 s), and final extension (72 °C for 10 min). A negative control was included in each run by replacing the template DNA with sterile water. The amplicons were examined through electrophoresis on a 2% agarose gel containing DNA binding fluorescent dye (SMOBIO Technology Inc., Hsinchu, Taiwan). For the gel electrophoresis of a highly suspected viral uveitis case with an ambiguous finding (a weak signal band or double bands) or a negative result, an on-demanded real-time PCR assay was used for discrepant analysis. Cobas Z480 (Roche) and 3 commercial kits (Cat. no 40–0378-32, 40–0562-32, and 40–0186-32; LightMix®, TIB Molbiol, Berlin, Germany) were used for detecting HSV, CMV, and EBV, while LightCycler 480 (Roche) and a commercial kit (Cat. no 40–0211-32; LightMix®, TIB Molbiol, Berlin, Germany) were used for detecting VZV.

### Statistical analysis

In descriptive analyses, quantitative variables were shown as mean ± standard deviation, and categorical data represented as numbers and percentages. Continuous variables between two groups were compared using Student’s t-test. The chi-square statistic and Fisher exact test were used to compare categorical variables as appropriate. *P* values adjusted by the Bonferroni post-hoc statistical analysis were calculated in the lens status category. A *P* value of < 0.05 was considered to be significant. Statistical analysis was performed using SPSS 20.0 (SPSS Inc., Chicago, Illinois, USA).

## Results

During this period of time, 102 eyes were eligible for inclusion in the study. There were 42 eyes that were herpesviridae-positive, including 9 HSV-AU eyes (8.8%), 5 VZV-AU eyes (4.9%), 27 CMV-AU eyes (26.5%), and 1 EBV-AU eye (1%). There were 60 eyes (58.8%) that were herpesviridae-negative on PCR analysis.

Comparison between herpesviridae-positive and herpesviridae-negative eyes showed that herpesviridae-positive patients were more likely to be male and have glaucoma (*P* = 0.008 and *P* = 0.047, respectively) on univariate analysis (Table 1). There was no significant difference in any of the other clinical features between herpesviridae-positive and negative eyes. There was also no difference between the two groups in the relative endothelial cell loss and cell count.

**Table 1:** Comparison of demographics and clinical features of herpesviridae-positive and herpesviridae-negative eyes

	Herpesviridae-Positive (42 eyes)	Herpesviridae-Negative (60 eyes)	Odds Ratio (95% Confidence Interval)	*P* value
Mean age (yrs)	61.26 ± 13.23	58.55 ± 11.95		0.292
Male Gender, no. (%)	36 (85.7)	37 (61.7)	3.731 (1.36 to 10.2)	0.008
Unilaterality, no. (%)	38 (90.5)	58 (96.7)		0.157
Visual acuity (logMAR)	0.84 ± 0.74	0.65 ± 0.36		0.132
IOP^a^ at presentation (mmHg)	23.99 ± 10.04	25.18 ± 12.57		0.610
Cornea edema, no. (%)	26 (61.9)	38 (63.3)		0.883
Keratic precipitates, no. (%)	37 (88.1)	46 (76.7)		0.145
Iris atrophy, no. (%)	5 (11.9)	10 (16.7)		0.504
Lens				0.140
Normal, no. (%)	13 (31)	21 (35)		
Cataract, no. (%)	11 (26)	24 (40)		
PCIOL^b^, no. (%)	18 (43)	15 (25)		
Glaucoma, no. (%)	15 (35.7)	11 (18.3)	2.475 (0.997 to 6.141)	0.047
Recurrence, no. (%)	19 (45.2)	21 (35)		0.297
Endothelial cells of lesion eye (no.)	1876.60 ± 673.62 (15)	1999.35 ± 373.76 (17)		0.522
Endothelial cell lost (no.)	779 ± 772.90 (14)	537.24 ± 103.80 (17)		0.279

^a^Intraocular pressure, ^b^posterior chamber intraocular lens

When comparing between CMV-positive and herpesviridae-negative cases, lens status and glaucoma were significant on univariate analysis (*P* = 0.021 and *P* = 0.011, respectively) (Table 2). After conducting Bonferroni post-hoc analyses between CMV-positive and herpesviridae-negative cases, we found a predominance of pseudophakic eyes in the CMV-positive group, as there was a significant difference between patients with normal lens versus pseudophakic eyes and patients with cataract versus pseudophakic eyes (*P* = 0.045 and *P* = 0.045, respectively) (Table 2). No significant difference was found between patients who had normal lens and patients with cataract. There was no difference in the endothelial cell count and relative endothelial cell loss of the eyes in the CMV-positive and herpesviridae-negative groups.

**Table 2:** Comparison of demographics and clinical features of CMV-positive and herpesviridae-negative eyes

	CMV-Positive (27 eyes)	Herpesviridae-Negative (60 eyes)	Odds Ratio (95% Confidence Interval)	*P* value
Mean age (yrs)	61.63 ± 11.81	58.55 ± 11.95		0.268
Male Gender, no. (%)	22 (81.5)	37 (61.7)		0.067
Unilaterality, no. (%)	25 (92.6)	58 (96.7)		0.231
Visual acuity (logMAR)	0.72 ± 0.37	0.65 ± 0.36		0.408
IOP^a^ at presentation (mmHg)	26.01 ± 9.35	25.18 ± 12.57		0.759
Cornea edema, no. (%)	14 (51.9)	38 (63.3)		0.312
Keratic precipitates, no. (%)	22 (81.5)	46 (76.7)		0.615
Iris atrophy, no. (%)	4 (14.8)	10 (16.7)		1.0
Lens				0.021^*^
Normal, no. (%)	6 (22)	21 (35)		
Cataract, no. (%)	6 (22)	24 (40)		
PCIOL^b^, no. (%)	15 (56)	15 (25)		
Glaucoma, no. (%)	12 (44.4)	11 (18.3)	3.564 (1.308 to 9.706)	0.011
Recurrence, no. (%)	13 (48.1)	21 (35)		0.245
Endothelial cells of lesion eye (no.)	1673.11 ± 731.16 (9)	2036.41 ± 314.63 (17)		0.087
Endothelial cell lost (no.)	880.78 ± 882.24 (9)	537.24 ± 427.97 (17)		0.190

^*^Comparison between lens status (Bonferroni post-hoc test): Normal vs. Cataract: *P* = 0.837, Normal vs. PCIOL: ***P*** **= 0.045,** Cataract vs. PCIOL: ***P*** **= 0.045**

^a^intraocular pressure, ^b^posterior chamber intraocular lens

## Discussion

Herpetic ocular inflammatory disease in immunocompetent patients is increasingly being implicated as a cause of what previously had been considered to be idiopathic [15, 17]. This has raised concern, especially CMV anterior uveitis, which is considered a newly recognized uveitis entity in immunocompetent patients [17]. Bloch-Michel et al. first described the pathogenic role of CMV in PSS after demonstrating local production of CMV-specific antibodies in the aqueous humor in 1987 [28]. CMV was later proved to be one of the etiologic agents of uveitic entities in immunocompetent patients [12, 17, 29, 30].

In our study, about 40% of non-HLA associated AU with increased IOP or corneal edema cases were herpesviridae-positive on PCR analysis. CMV was the most frequent identified virus in herpetic AU patients. About two-thirds of our patients were found to be CMV-positive in the herpesviridae-positive anterior uveitis group. Our findings correspond well to previous reports, which described that the proportion of CMV-associated AU is about two-thirds of the patients with viral AU in Singapore and Thailand [12, 17]; Japan reported a lower rate at 39% [20]. In contrast, in an Italian population, HSV (83.3%) was the most common cause of viral AU, followed by VZV (11.9%) and CMV (4.8%) [19]. In a tertiary referral center in Virginia, USA, Engelhard et al found that of viral AU, the most prevalent pathogen was HSV (51.3%), followed by VZV (43.6%) and CMV (2.6%) [31]. However, the designs and inclusion criteria were different between studies. We had excluded herpetic epitheliopathy cases, so the percentage of CMV might be considerably higher in our study than that reported in other studies. Worldwide, the estimated seroprevalence of CMV in the general population is 45 to 100%, higher in Asia in contrast to Western countries [32]. However, populations in Italy (77%) and Sweden (83%) have slightly higher CMV seroprevalence than other countries in Western Europe [33, 34]. In Asian population, the seroprevalence of CMV in Taiwan, Singapore, and Japan is 91.1, 87, and 69.1%, respectively [35–37]. Therefore, it has been speculated that the high rate for CMV AU infections in our population may be due to the increased latent systemic CMV infections in Asians [17].

In our series, we found that herpesviridae-positive patients are more likely to be male, and have glaucoma at diagnosis. However, the other clinical features are similar between the herpesviridae-positive and herpesviridae-negative eyes (Table 1). Moreover, there were no clinical features that could enable the physician to differentiate between the CMV-positive and herpesviridae-negative eyes (Table 2). In the report of Chee SP et al, the study also mentioned that CMV-positive and CMV-negative PSS eyes were clinically similar. Furthermore, they found that one-third of the eyes with presumed PSS and FHI were positive for CMV-DNA on PCR analysis of their aqueous [10]. This may be due to the reason that ocular manifestations of a viral infection is probably determined by the host’s genetic make-up as well as the immune status of that eye, and may not be specific to a certain virus [17]. The herpesviridae-negative results may be attributed to non-viral disease or elimination of viral DNA upon the aqueous humor tapping time. The latter could be a result of the rapid acute ocular inflammation course, which could be seen from the quick rise in IOP and self-limiting tendency of the disease [10, 12].

It is important to distinguish whether an anterior uveitis is caused by viral infection or not as treatment with immunosuppressants alone without appropriate antiviral therapy could possibly worsen the infection and potentially cause further ocular damage [11]. However, until now, viral isolation, serum antibody titers, or PCR analysis is required to make the diagnosis of CMV AU. Both viral isolation and serum antibody titers require days to weeks for completion, which may delay diagnosis and affect treatment choices [15]. PCR analysis is a common and popular laboratory technique which can be a more practical tool [21].

A significant proportion of our patients diagnosed with CMV-positive anterior uveitis had undergone intraocular lens implantation. Complicated cataract secondary to recurrent uveitis might be a contributing factor. On the other hand, Zamir et al first reported a case of CMV endotheliitis triggered by cataract surgery [38]. Two other patients in Spain who developed CMV anterior uveitis after cataract extraction were also recorded [9]. It is difficult to determine whether CMV anterior uveitis has occurred due to a newly acquired infection, or if it was due to surgery-driven reactivation of a previous asymptomatic ocular infection [9]. One possible factor for the increase of CMV-positive AU in pseudophakic eyes may be the result of local use of immunosuppressive medication after cataract surgery [39]. Another possible factor may be due to blood-aqueous barrier disruption after a cataract extraction [40]. Previous studies indicated that the blood–aqueous barrier (BAB) reaction is reduced after phacoemulsification due to inflammatory mediators such as prostaglandin E2, causing disruption of the BAB by breaking up the tight junctions in the nonpigmented ciliary epithelium [41–43]. However, further studies are needed to provide better insights into the inflammatory response that may potentiate CMV anterior segment infection.

## Conclusions

Our study shows that CMV is an important cause of non-HLA anterior uveitis among the Chinese population in Taiwan, with a wide spectrum of clinical manifestations. Last but not least, PCR analysis of the anterior chamber fluid is important for the confirmation of the diagnosis of viral anterior uveitis.

## Abbreviations

AAU: Acute anterior uveitis
AU: Anterior uveitis
BAB: Blood–aqueous barrier
CMV: Cytomegalovirus
EBV: Epstein-Barr virus
FHI: Fuchs’ heterochromic iridocyclitis
HSV: Herpes simplex virus
IOP: Intraocular pressure
PCR: Polymerase chain reaction
PSS: Posner-Schlossman syndrome
VZV: Varicella zoster virus
